# Physiological Response of Soybean Plants to Water Deficit

**DOI:** 10.3389/fpls.2021.809692

**Published:** 2022-01-31

**Authors:** Xiyue Wang, Zihao Wu, Qi Zhou, Xin Wang, Shuang Song, Shoukun Dong

**Affiliations:** Agronomy College, Northeast Agricultural University, Harbin, China

**Keywords:** soybean, drought stress, protective enzyme, soil moisture content, plant physiology

## Abstract

Soybean is an important cash crop in the world, and drought is the main reason for the loss of soybean plants productivity, with drought stress during the most water-sensitive flowering period of soybeans. In this article, drought-tolerant variety Heinong 44 (HN44) and drought-sensitive variety Heinong 65 (HN65) were used as experimental materials. Drought treatment was carried out at the early flowering stage. The method of controlling soil moisture content was used to simulate different degrees of drought, and the physiological changes of these two varieties of soybean under different soil moisture contents were studied. The results showed that with a decrease in soil moisture content, the content of malondialdehyde (MDA) in soybean leaves increased significantly; the activities of peroxidase (POD), catalase (CAT), and ascorbic acid peroxidase (APX) increased first and then decreased; the content of proline, soluble sugar, and soluble protein increased; and the total antioxidant capacity (T-AOC) increased significantly. When the soil moisture content was 15.5%, the degree of membrane lipid peroxidation, osmotic regulatory substances, antioxidant enzyme activity, and T-AOC increased the most, and the decrease in drought-tolerant variety HN44 was significantly less than that of drought-sensitive variety HN65. Our research reveals the response law of soybean crops to physiological characteristics under water deficit and provides theoretical basis and guiding significance for drought-resistant cultivation and breeding of soybean.

## Introduction

A native to Chine and grown throughout the country, soybean is an important grain and oil crop that plays an important role in food, pharmaceutical, and feed processing and bioenergy production (Zhang Y. F. et al., 2019). However, the root system of soybeans is underdeveloped, while water demand during the plant life cycle is high. Indeed, the root system is more sensitive to adverse conditions, such as drought, which may cause yield losses ranging from 25 to 50% (Zou et al., 2020). Furthermore, the soybean plant is most sensitive to water deficit during the flowering period, when water shortage will most seriously affect yield. Therefore, enhancing drought resistance of the soybean root system during the flowering period has become a major focus of research.

China has vast arid and semiarid areas that account for 52.5% of the total land area in the country (Zhao et al., 2017). In these areas, agricultural drought, which refers to limited soil-water availability due to insufficient precipitation over a period of time, causes a decrease in soil moisture content to a level that crop plant growth and development is severely hindered, thereby reducing crop yield (Jiang et al., 2019). The impact of drought on crop yield is one of the main limiting factors in soybean production. Thus, the reduction caused by drought in annual total crop production accounts for over 50% of all yield losses caused by all stress factors together. In recent decades, the drought-prone area in China has been increasing. In addition, drought frequency is on the rise, and disaster events have been steadily increasing. Further, future climate projections all coincide in an increasing trend in ambient temperature, which will undoubtedly increase evapotranspiration, making drought-related reductions in crop yields even worse (Jia et al., 2019). However, some modern technologies have been developed to assess the increased drought stress in soybean and other plant species (Kovar et al., 2019; Basal and Szabó, 2020; Botyanszka et al., 2020), thus aiming to better understand the effects of drought stress on plants.

Drought stress is caused by the scarcity of long-term precipitation and lack of irrigation water, leading to reduced water supply to the soil tillage layer for a long period and, consequently, to an effective water deficit for crop absorption and utilization. In such conditions, it is difficult for crop roots to absorb sufficient amounts of water, which, coupled with an excessive evaporative demand, interferes with normal physiological activity (Xu et al., 2018). Under normal conditions, reactive oxygen species (ROS) produced in plant cells are countered by the clearance systems they have evolved for that purpose (Wang H. H. et al., 2014). To maintain normal plant metabolism, plants remove excess ROS through intracellular antioxidant enzyme activity to alleviate the potential harmful effects of drought stress on normal plant metabolism (Gill and Tuteja, 2010; Das and Roychoudhury, 2014; Feng et al., 2020). Thus, for example, studies have shown that drought stress can induce the accumulation of ROS, causing increased membrane lipid peroxidation. Furthermore, drought stress can reduce seed germination rate, damage photosynthetic organs, and reduce plant height, pod number, and yield (Kausar et al., 2012).

Under arid conditions, soybean plants lose water content, thus causing leaves to wilt and droop, unbalancing plant water status, and decreasing the cell water potential and turgor (Zhang et al., 2020). If the plant is subjected to dry conditions for a long enough period, the phenomenon of cell water loss is aggravated, causing irreversible damage to the plant tissues, which ultimately leads to plant death (Shen et al., 2017). To resist potential damage, plants have evolved mechanisms to adapt to adverse environments in the long term and undergo physical, physiological, and biochemical changes to adapt to stress and successfully complete their life cycle (Yang et al., 2007). Worldwide, numerous studies have been conducted on plant morphology and physiological and biochemical characteristics in order to explore the drought-resistant potential of soybeans to improve plant drought resistance (Ma et al., 2012; Jiang et al., 2014; Wu et al., 2014; Chimungu et al., 2015).

In this experiment, two spring soybean varieties contrasting for drought resistance, Heinong 44 and Heinong 65, were subjected to a water-deficit gradient during the most sensitive period of flowering in soybeans. Osmotic adjustment and antioxidant activity were explored under different soil moisture content conditions to provide a theoretical basis and guidance for drought-resistant cultivation and breeding of soybeans (Zhang K. et al., 2019).

## Materials and Methods

### Plant Materials and Equipment

Soybean varieties included Heinong 44 (HN44, drought-resistant variety) and Heinong 65 (HN65, drought-susceptible variety). Soybeans were identified and screened for in this study (Wang et al., 2012).

The experiment was conducted using soil from a cultivated field in Northeast Heilongjiang Province. The basic fertility characteristics of the soil at the study site are summarized in Table 1.

**TABLE 1:** Base fertility of tested black soil.

Total Nitrogen g/kg	Total Phosphorus g/kg	Total Potassium g/kg	Nitrate Nitrogen mg/kg	NH_4_^+^N mg/kg	Available Potassium mg/kg	Available Phosphorus mg/kg	Organic Matter g/kg
1.44	0.65	17.60	45.21	17.31	160.12	22.43	25.56

The experimental equipment included a glass rain shelter, a plastic barrel (bottom hole, 33 cm, inner diameter 28 cm), and an EM-50 moisture meter buried approximately 10 cm deep in the soil.

### Experimental Design

This was a pot experiment in which plastic buckets with a load of 13 kg of soil were used to grow three soybean plants per pot. Soil moisture content was monitored using an EM-50 moisture meter. Soil water content at field capacity was 42.8%. To keep the experimental period the same in all treatments while subjecting plants in each treatment group to a different level of water deficit, the specific water treatments were as follows: unlimited soil moisture was maintained in all pots before the R1 period; then, starting from the R1 period, (1) water was continuously withheld from the first group for 7 days; (2) water was withheld from the second group for 1 day, followed by rewatering for 6 days; (3) water was withheld from the third group for 2 days, followed by rewatering for 5 days; (4) water was withheld from the fourth group for 3 days, followed by rewatering for 4 days; (5) water was withheld from the fifth group for 4 days, followed by rewatering for 3 days; (6) water was withheld from the sixth group for 5 days, followed by rewatering for 2 days; and (7) water was withheld from the seventh group for 6 days, followed by rewatering for 1 day. The eighth group served as the control (CK) group, which was continuously watered throughout the experimental period. Upon completion of the experimental 7 days period, the following groups were compared: 0 days without water represented no water deficit (31.5% soil moisture content); 1 and 2 days without water represented mild water deficit (24.1% and 22.3% soil moisture content, respectively); 3 and 4 days without water represented moderate water deficit (20.1% and 20.8% soil moisture content, respectively); and 5, 6, and 7 days without water represented severe water deficit (15.5%, 14.7%, and 14.5% soil moisture content, respectively). Eight treatments were administered. After completing the experiment, two to three leaf samples were collected from each pot at 8:00–9:00 h. Per treatment process was repeated five times. Samples were frozen in liquid nitrogen and stored in an ultralow-temperature refrigerator until analysis.

### Analytical Methods

#### Determination of Osmotic Adjustment

##### Determination of Proline: Acid Triketone Method

First, leaf samples (0.1 g) were placed in centrifuge tubes; then, 1 ml of 3% sulfosalicylic acid solution was added to each tube for extraction in a boiling water bath for 10 min. After cooling to room temperature, the tubes were centrifuged at 12,000 rpm for 10 min; then, 0.4 ml of the supernatant was withdrawn and added with 0.4 ml of glacial acetic acid and 0.6 ml of chromogenic solution. This mixture was heated in a boiling water bath for 40 min and then cooled before 1 ml of toluene was added to fully extract the red substance. After stratification, the toluene layer was withdrawn and compared at 520 nm using a spectrophotometer (adjusted to zero with toluene). Proline concentration in the reaction volume was determined from a standard curve, and proline content in the sample was calculated according to the following formula:


$$P\,r\,o\,l\,i\,n\,e\,\left(\mu \,g\cdot g^{-1}\cdot F\,W\right)=\left(C\times V/A\right)/W$$

where *C* is the proline content (μg) in the extract as calculated from the standard curve; *V* is the total volume of extract (ml); *A* is the volume (ml) absorbed during measurement; and *W* is the sample weight (g).

##### Determination of Soluble Sugar Content: Anthrone Method

Referring to the experimental method of Pan et al. (2012). Fresh 0.1-g leaf samples were placed in centrifuge tubes, added with 1 ml distilled water, and centrifuged for 10 min at 12,000 rpm. Then, 0.2 ml of sample extracts or distilled water for the control were added to 1-ml anthrone reagent, shaken well, boiled in a boiling water bath for 10 min, removed, and cooled, and absorbance was measured at 620 nm using a spectrophotometer. The zero point was adjusted to a blank, and the absorbance value was recorded. The micrograms of the corresponding sugar were determined using a standard curve.


Solublesugarcontent(%)=sugarcontent(g)×dilution(10-fold)/{sampleweight(g)×106}×100.

##### Determination of Soluble Protein: Coomassie Brilliant Blue Method

Referring to the experimental method of Li (2000). Fresh leaf samples (0.1 g) were weighed, and a small amount of quartz sand was added to the homogenates and 1 ml distilled water was added to each before centrifuging at 12,000 rpm for 10 min. The sample extracts (0.2 ml) were placed in test tubes (two repeat tubes) and added with 1 ml Coomassie brilliant blue G-250 reagent; the mixture was shaken vigorously, and after 2 min, absorbance was measured at 595 nm. Soluble protein content was calculated using the corresponding standard curve obtained using bovine serum albumin (BSA).

#### Determination of Membrane Lipid Peroxidation

##### Determination of Malondialdehyde: Colorimetric Method

Referring to the experimental method of Li (2000). Leaf samples (0.1 g) were added with 10% trichloroacetic acid (TCA) 1 ml (total), ground in a mortar, and centrifuged at 12,000 rpm for 10 min. Then, 0.2 ml of 0.67% thiobarbituric acid (TBA) was added to 0.4 ml of each homogenate before mixing and boiling in a water bath at 100°C for 30 min; after cooling, the samples were centrifuged again. The absorbance values of the supernatants were measured at 450, 532, and 600 nm, and the malondialdehyde (MDA) concentration was calculated according to the following formula and then MDA content was calculated on a fresh weight basis. CMDA = 6.45 (*A*532 − *A*600) − 0.56*A*450 (μmol/L).

#### Determination of Antioxidant Enzyme Activity

##### Peroxidase (POD) Activity Determination: Colorimetric Method (Zhang, 2004)

Referring to the experimental method of Zhang (2004). To prepare the reaction solution, 200 ml phosphate-buffered saline (PBS) (0.2 M, pH 6.0) were mixed with 0.076 ml liquid (original liquid) guaiacol (2-methoxyphenol) and stirred until dissolved; after cooling, 0.112 ml of 30% H_2_O_2_ were added, and the solution was mixed well before storing in a refrigerator. This reaction solution (3 ml) was added to 30 μl of enzyme solution and PBS as a control and then OD_470_ was determined (1 min). One enzyme activity unit (u) was defined as a 0.01 increase in OD_470_ per minute.


POD=(ΔA470×V)t/(W×V×s0.01×t)(μg/min)

where Δ*A*_470_ is the change in absorbance with reaction time, *W* is the sample fresh weight (g), *t* is the reaction time (min), *V*_t_ is the total volume of the reaction (3.3 ml), and *V*_s_ is the volume (0.03 ml) of enzyme extract used in the reaction.

##### Catalase Activity Assay: Colorimetric Assay

Referring to the experimental method of Zhang (2004). To prepare the reaction solution, 200 ml PBS (0.15 M, pH 7.0) were added with 0.3092 ml of 30% H_2_O_2_ and shaken vigorously. For enzyme activity analysis, 3 ml of the reaction solution were added to 0.1 ml (adjusted according to the situation) of enzyme extract, with PBS as the zero control. determination of OD_240_ (UV) (reading every 1 min for a total of 2 min). One unit (u) of enzyme activity was calculated as a 0.01 decrease in OD_240_ per minute.


CAT=[ΔA×240V]t/(W×V×s0.01)(U/gmin)

ΔA=240(A-240⁢iA)240⁢f/(t-ft)i

where *A*_240f_ and *A*_240i_ are the final and initial *A*_240_ values, respectively; *t*_f_ is the reaction final time; *t*_i_ is the reaction initial time; *W* is the sample fresh weight (g); *t* is the reaction time (min); *V*_t_ is the total volume of the reaction (3.1 ml); and *V*_s_ is the volume (0.1 ml) of enzyme extract in the reaction.

##### Determination of Ascorbic Acid Peroxidase (APX) Activity: Colorimetric Method

Referring to the experimental method of Zhang (2004). Take 0.10-ml enzyme solution, add 2.60-ml PBS containing 0.1-mM EDTA-Na_2_ (0.05 mol/L, pH 7.0), then add 0.15 ml of 5 mM AsA, and finally 0.15 ml of 20 mM H_2_O_2_ were added; then, immediately the change of OD_290_ was measured every 1 min at 20°C two times. One enzyme activity unit (u) was defined as a 0.01 change in A_290_ per min. Enzyme activity was expressed as U/g (FW).


APXactivity(U/g)=△A×290V/t0.01×V×st×W

where △*A*_290_ indicates the change in absorbance at 290 nm, *V*_t_ is the total extraction volume, *V*_s_ is the volume of enzyme solution, *W* is the leaf fresh weight, and *t* is the reaction time.


VC-PODactivity(mmolVCgFW-1⋅h)-1=(△A*V×tV×60×1,000)/(FW×V×s2.8×t)

where *V*_t_ is the total volume (ml) of enzyme solution; FW is the sample fresh weight (g); *V*_s_ is the volume of enzyme extract (ml) in the reaction; *V* is the reaction volume (ml); △*A* is the change in A_290_ in 1 min; *t* is the reaction time (min); and 2.8 is the millimolar extinction coefficient of VC (2.8 mM/cm).

#### Determination of Total Antioxidant Capacity

Determination of total antioxidant capacity (T-AOC) was performed using the corresponding commercial kit (Suzhou Keming Biotechnology Co., Ltd.) according to the instructions of the manufacturer.

### Data Analysis

The data were processed and analyzed using SPSS version 17.0 and Origin version 9.

## Results

### Effects of Water Deficit on Membrane Lipid Peroxidation in Soybean Leaves

**FIGURE 1:**
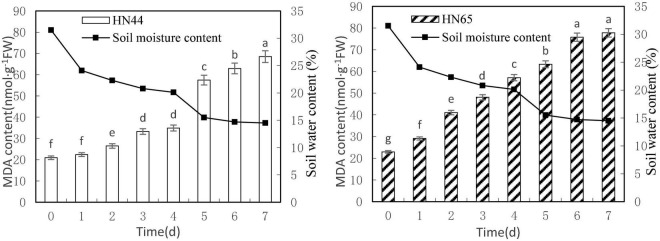
Effect on MDA content under different soil water contents. Indicates that the values marked with different letters are significantly different, and those marked with the same letter are considered to be statistically insignificant.

Figure 1 shows that MDA content in the tested genotypes, HN44 and HN65, steadily increased with decreasing soil moisture content from 31.5 to 14.5%. MDA content peaked in both varieties at 7 days of treatment, with 68.51 nmol/g in HN44 and 77.78 nmol/g in HN65. Furthermore, MDA content in HN44 was not significantly different between 0 and up to 4 days without watering, but it did differ significantly among the rest of the treatments. In contrast, MDA content did not significantly differ in HN65 between 6 and 7 days without watering, but it did among the rest of the treatments.

Table 2 shows that MDA content in HN44 soybean leaves increased from 7.04 to 25.74% upon withholding water for 1–3 days. When the soil moisture content was 15.5% (i.e., water withheld for 5 days), MDA content in HN44 increased by the largest proportion, to 64.59%. In turn, HN65 exhibited the largest increase in MDA content from 22.3 to 40.95% after 4 days without water, at which time soil moisture content was 20.1%. In contrast, MDA content in HN44 increased the least, to 4.88% on the fourth day after withholding water, while HN65 showed the smallest increase in MDA content after 7 days of withholding water, when soil moisture content had fallen to 14.5%. Overall, when HN44 and HN65 experienced soil moisture contents of 15.5% and 22.3%, respectively, membrane lipid peroxidation in soybean leaves was high, the degree of injury was severe, and as water-deficit treatment was extended, the degree of damage increased correspondingly.

**TABLE 2:** Effect of drought on the relative increase rate of MDA content (%).

Varieties	0 31.5%	1 24.1%	2 22.3%	3 20.8%	4 20.1%	5 15.5%	6 14.7%	7 14.5%
HN44	0.0	7.04	18.10	25.74	4.88	64.59	9.54	8.86
HN65	0.0	27.00	40.95	17.07	18.86	10.79	19.72	2.64

*The chart shows the relative increase rate of the next day compared with the previous day.*

### Effects of Water Deficit on Osmotic Adjustment in Soybean Leaves

#### Effects on Proline Content

**FIGURE 2:**
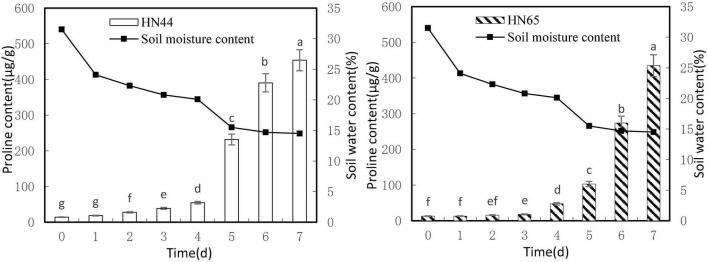
Effect on proline content under different soil water contents. Indicates that the values marked with different letters are significantly different, and those marked with the same letter are considered to be statistically insignificant.

Figure 2 shows that leaf proline content increased as soil moisture decreased from 31.5 to 14.5%. Both varieties reached a maximum proline content at 7 days of water-withholding treatment, with HN44 at 453.95 μg/g and HN65 at 434.73 μg/g. No significant differences in proline content in HN44 were noted between drought 0 and 1 day of withholding water, but significant differences were noted among the rest of the treatments. In turn, proline content in HN65 was not significantly different up to 2 days of withholding water nor between 2 and 3 days of treatment. However, the remaining treatments differed significantly from one another.

Table 3 shows that proline content in HN44 soybean leaves first increased and then decreased within the 31.5–50.52% range. When the soil moisture content was 15.5%, that is, after 5 days of withholding water, proline content in HN44 increased rapidly, and the maximum increase rate was 324.19%. When the soil moisture content was 14.7%, the largest increase in proline (at 166.10%) for HN65 was recorded at 6 days of withholding water. In contrast, when the soil moisture content was 14.5%, that is, after 7 days of treatment, the smallest increase in proline (16.23%) was observed in HN44. However, HN65 showed the smallest increase in proline (0.15%) at a soil moisture content of 24.1% after water had been withheld for 1 day. Thus, when the soil moisture content was 15.5% and 14.7% in HN44 and HN65 pots, respectively, damage to soybean leaves significantly increased. Furthermore, proline content significantly increased, presumably to reduce water-deficit-induced damage, and the response of the drought-resistant variety was significantly faster than that of the drought-sensitive variety.

**TABLE 3:** Effect of drought on the relative growth rate of proline content (%).

Varieties	0 (31.5%)	1 (24.1%)	2 (22.3%)	3 (20.8%)	4 (20.1%)	5 (15.5%)	6 (14.7%)	7 (14.5%)
HN44	0.0	31.55	50.52	40.32	40.60	324.19	68.60	16.23
HN65	0.0	0.15	19.53	15.38	162.61	116.79	166.10	58.72

*The chart shows the relative increase rate of the next day compared with the previous day.*

#### Effects of Water Deficit on Soluble Sugar Content in Soybean Leaves

**FIGURE 3:**
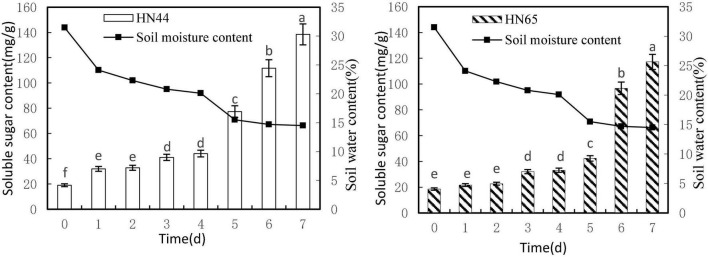
Effect on soluble sugar content under different soil water contents. Indicates that the values marked with different letters are significantly different, and those marked with the same letter are considered to be statistically insignificant.

Figure 3 shows that soluble sugar content increased as soil moisture decreased from 31.5 to 14.5%. Both varieties reached a maximum soluble sugar content at 7 days of water-withholding treatment, with HN44 at 138.49 mg/g and HN65 at 117.18 mg/g. Soluble sugar content in HN44 did not differ significantly between 1 and 2 days nor between 3 and 4 days of the water-deficit treatment, whereas significant differences were noted among the rest of the treatments. As for HN65, there were no significant differences in soluble sugar content between 0 and 2 days as well as between 3 and 4 days of water-withholding treatment. However, significant differences were noted among the remaining treatments.

Table 4 shows that soluble sugar content in HN44 increased the most when the soil moisture content was 15.5%, at 5 days of treatment, reaching 75.33%. In contrast, HN65 showed the largest increase (128.49%) at a soil moisture content of 14.7%, at 6 days from treatment initiation; when the soil moisture content was 22.3%, at 2 days of treatment, soluble sugar content in HN44 increased by 2.69%, while in HN65, it increased by 3.37% at a soil moisture content of 20.1%, at 4 days after water treatment initiation. When the soil moisture content was 15.5% and 14.7% in HN44 and HN65, respectively, damage to soybean leaves was significantly increased. Under these conditions, soluble sugar content increased significantly, presumably to reduce water-deficit-induced damage. Furthermore, the response of drought-resistant variety was significantly higher than that of the drought-sensitive variety.

**TABLE 4:** Effect of drought on the relative growth rate of soluble sugar content (%).

Varieties	0 (31.5%)	1 (24.1%)	2 (22.3%)	3 (20.8%)	4 (20.1%)	5 (15.5%)	6 (14.7%)	7 (14.5%)
HN44	0.0	67.93	2.69	25.11	7.32	75.33	44.45	23.96
HN65	0.0	16.83	4.80	41.23	3.37	27.73	128.49	21.15

*The chart shows the relative increase rate of the next day compared with the previous day.*

#### Effects of Water Deficit on Soluble Protein Content in Soybean Leaves

**FIGURE 4:**
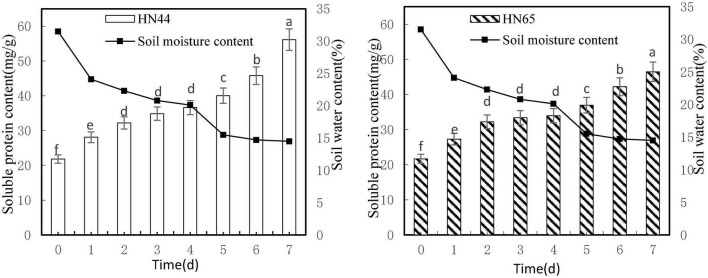
Effect on soluble protein content under different soil water contents. Indicates that the values marked with different letters are significantly different, and those marked with the same letter are considered to be statistically insignificant.

Figure 4 shows that soluble protein content increased as soil moisture decreased from 31.5 to 14.5%. Both varieties reached a maximum soluble protein content at 7 days from treatment initiation, with HN44 peaking at 56.14 mg/g soluble protein and HN65 peaking at 46.46 mg/g soluble protein. Furthermore, soluble protein contents were not significantly different in either variety from 2 to 4 days from treatment initiation, although significant differences were observed between HN44 and HN65 in the other water-deficit treatments.

As shown in Table 5, from day 1 to day 4 after withholding water supply, soluble protein content in HN44 and HN65 soybean leaves gradually increased. When the soil moisture content was 24.1%, soluble protein content in HN44 and HN65 increased the most after 1 day of withholding water, with HN44 reaching 28.69% and HN65 reaching 25.8%. Conversely, when the soil moisture content was 20.1%, at 4 days of water-withholding treatment, soluble protein content in HN44 and HN65 increased the least, namely, 4.96% in HN44 and 1.68% in HN65. When both HN44 and HN65 experienced 20.1% of soil moisture, damage to soybean leaves increased significantly, and soluble protein content greatly increased, presumably to mitigate the damage (Xu et al., 2020). Soluble protein content increased concomitant with the extension of the water-deficit period.

**TABLE 5:** Effect of drought on the relative growth rate of soluble protein content (%).

Varieties	0 (31.5%)	1 (24.1%)	2 (22.3%)	3 (20.8%)	4 (20.1%)	5 (15.5%)	6 (14.7%)	7 (14.5%)
HN44	0.0	28.69	14.71	8.26	4.96	9.40	14.34	22.63
HN65	0.0	25.80	18.20	3.66	1.68	8.80	14.21	10.09

*The chart shows the relative increase rate of the next day compared with the previous day.*

### Effects of Water Deficit on Antioxidant Enzyme Activity in Soybean Leaves

#### Effects on Peroxidase Activity

**FIGURE 5:**
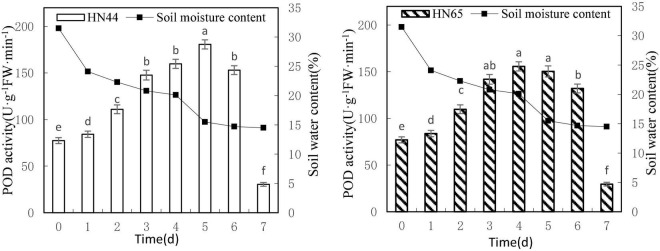
Effect on POD activity under different soil water contents. Indicates that the values marked with different letters are significantly different, and those marked with the same letter are considered to be statistically insignificant.

Figure 5 shows that POD activity tended to increase first and then decrease as soil moisture decreased from 31.5 to 14.5%. HN44 reached a maximum of 180.65 U/g min at 5 days, whereas HN65 reached a maximum of 155.71 U/g min, that is, 1 day earlier. At 7 days from treatment initiation, POD activity dropped rapidly to the lowest values over the experimental period, with 30.42 U/g min for HN44 and 29.57 U/g min for HN65. There were no significant differences in POD activity in HN44 among 3, 4, and 6 days of treatment, whereas significant differences were noted among the other treatments. No significant differences in POD activity were noted in HN65 between 3 and 6 days nor among 3, 4, and 5 days of treatment. In contrast, significant differences were noted among the remaining treatments.

Table 6 shows that POD activity increased from 8.81% at 1 day to 33.05% at 3 days of water treatment in HN44 soybean leaves. When the soil moisture content was 20.8% and water was withheld for 3 days, POD activity in HN44 peaked at 33.05%. In contrast, HN65 showed a maximum POD activity increase of 31.09% at 2 days of water-withholding treatment, when the soil moisture was at 22.3%. At 7 days from treatment initiation, POD activity decreased by 80.12% in the leaves of HN44 and by 77.61% in those of HN65. When the soil moisture content was 14.5%, after a 7 day period of withholding water, POD activity fell below the control levels. Furthermore, when the soil moisture content was 15.5% and 20.1% in HN44 and HN65, respectively, leaf damage significantly increased and POD exhibited the greatest ability to remove ROS.

**TABLE 6:** Effect of drought on the relative growth rate of POD activity (%).

Varieties	0 (31.5%)	1 (24.1%)	2 (22.3%)	3 (20.8%)	4 (20.1%)	5 (15.5%)	6 (14.7%)	7 (14.5%)
HN44	0.0	8.81	31.81	33.05	8.19	13.05	−15.28	−80.12
HN65	0.0	8.77	31.09	29.22	9.76	−3.46	−12.17	−77.61

*The chart shows the relative increase rate of the next day compared with the previous day.*

#### Effects of Water Deficit on Catalase Activity of Soybean

**FIGURE 6:**
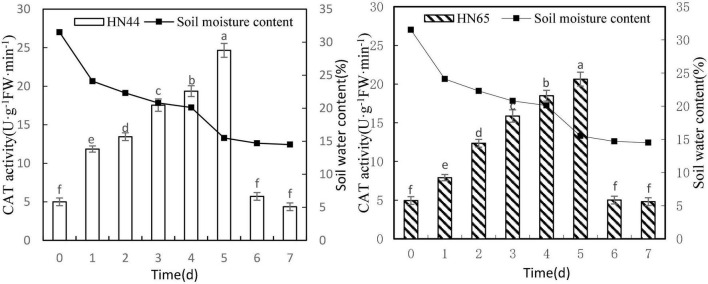
Effect on CAT activity under different soil water contents. Indicates that the values marked with different letters are significantly different, and those marked with the same letter are considered to be statistically insignificant.

Figure 6 shows that CAT activity tended to increase first and then decrease as the soil moisture content decreased from 31.5 to 14.5%. When the soil moisture content was 15.5%, at 5 days from treatment initiation, CAT activity reached a maximum in both varieties. Specifically, CAT activity reached 24.65 U/g min in HN44 and 20.64 U/g min in HN65. No significant difference in CAT activity in HN44 was noted among 0, 6, and 7 days, but significant differences were noted among the other treatments; furthermore, a very similar trend was observed for CAT activity in HN65.

As shown in Table 7, when the soil moisture content was 24.1% after 1 day of water-deficit treatment, CAT activity in the two varieties increased the most, with HN44 reaching 137% and HN65 reaching 59.8%. Conversely, CAT activity in the two varieties decreased the most when soil moisture content was 14.7%, at 6 days from treatment initiation. At this point, HN44 and HN65 decreased by 76.84% and 75.73%, respectively, and when the soil moisture content was 14.7–14.5%, at 6–7 days from treatment initiation, CAT activity was reduced to the control level. Lastly, when the soil moisture content was 15.5%, damage to soybean leaves significantly increased, and the ability of CAT to remove ROS reached the limit.

**TABLE 7:** Effect of drought on the relative growth rate of CAT activity (%).

Varieties	0 (31.5%)	1 (24.1%)	2 (22.3%)	3 (20.8%)	4 (20.1%)	5 (15.5%)	6 (14.7%)	7 (14.5%)
HN44	0.0	137.00	13.50	30.48	10.31	27.32	−76.84	−23.64
HN65	0.0	59.80	56.13	28.50	16.51	11.63	−75.73	−3.99

*The chart shows the relative increase rate of the next day compared with the previous day.*

#### Effects of Water Deficit on Activity of Ascorbic Acid Peroxidase in Soybean

**FIGURE 7:**
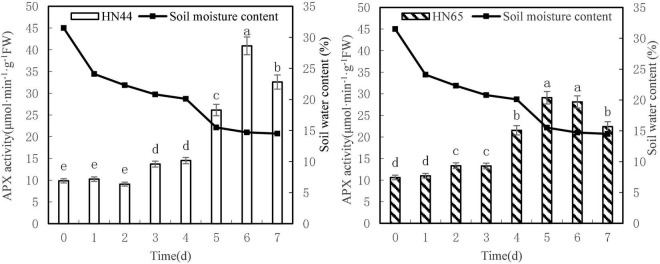
Effect on APX activities under different soil water contents. Indicates that the values marked with different letters are significantly different, and those marked with the same letter are considered to be statistically insignificant.

Figure 7 shows that APX activity tended to increase first and then decrease as soil moisture decreased from 31.5 to 4.5%. APX activity in HN44 reached a maximum of 40.90 U/g min at 6 days, whereas HN65 reached a maximum of 29.08 U/g min at 5 days after water treatment was initiated. APX activity in HN44 was not significantly different for 0 to 2 days nor between 3 and 4 days of treatment. However, significant differences were noted among the remaining treatments. Similarly, no significant differences in APX activity in HN65 were noted from 0 to 4 days, for 7 days, or between 5 and 6 days of treatment. However, significant differences were noted among the other treatments.

Table 8 shows that APX activity in HN65 soybean leaves decreased from 62.38 to 20.30% after 4–7 days of treatment. Furthermore, APX activity increased by 80.07% in HN44 when the soil moisture content was 15.5%, after 5 days of water deficit; meanwhile, the activity increased by 62.38% in HN65 at a soil moisture content of 20.1%, after 4 days of water-deficit treatment. When the soil moisture content was 14.5% after 7 days water was withheld, APX activity decreased by 20.35% in HN44 and by 20.30% in HN65. When the soil moisture content was 14.7% in pots sown with HN44 and 15.5% in pots sown with HN65, damage to soybean leaves significantly increased, and the ability of APX to remove ROS was enhanced.

**TABLE 8:** Effect of drought on the relative growth rate of APX activity (%).

Varieties	0 (31.5%)	1 (24.1%)	2 (22.3%)	3 (20.8%)	4 (20.1%)	5 (15.5%)	6 (14.7%)	7 (14.5%)
HN44	0.0	3.95	−11.26	50.80	5.90	80.07	56.51	−20.35
HN65	0.0	3.69	21.15	−0.47	62.38	34.99	−3.31	−20.30

*The chart shows the relative increase rate on the next day compared with the previous day.*

### Effects of Water Deficit on the Total Antioxidant Capacity of Soybean

**FIGURE 8:**
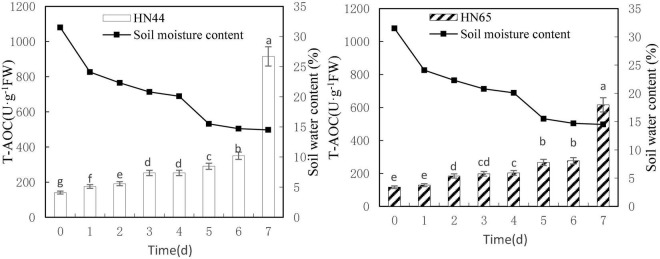
Effect on T-AOC under different soil water contents. Indicates that the values marked with different letters are significantly different, and those marked with the same letter are considered to be statistically insignificant.

Figure 8 shows that T-AOC of HN44 and HN65 generally increased with decreasing soil moisture content from 31.5 to 14.5%. Both varieties reached a maximum value at 7 days (915.55 U/g in HN44 and 77.78 U/g in HN65) after water was withheld. No significant difference in T-AOC of HN44 was noted between 3 and 4 days of treatments, but significant differences were noted among the other treatments. No significant differences in T-AOC of HN65 were observed when water was withheld from 0 to 2, 3 to 4, or 5 to 6 days. However, significant differences were noted among the other treatments.

Table 9 shows that the increase in T-AOC of HN44 soybean leaves from 0.06 to 161.5% under water-deficit conditions lasting 4–7 days. When the soil moisture content was 14.5% after 7 days without water, T-AOC of the two varieties increased the most. Specifically, HN44 reached 161.5%, and HN65 reached 122.8%. Conversely, T-AOC of the two varieties increased the least, namely, 0.06% for HN44 and 2.82% for HN65 when the soil moisture content was 20.1% after 4 days without water. During the 7 day treatment without water, T-AOC increased rapidly in the two varieties tested. Thus, T-AOC increased 2.61-fold in HN44 compared with that at 6 days, and T-AOC increased 2.23-fold in HN65 compared with that at 6 days. At 14.5% soil water content, the antioxidant capacity of cells increased, and the ability to remove ROS increased, thus achieving a protective effect on plants.

**TABLE 9:** Effect of drought on the relative growth rate of T-AOC (%).

Varieties	0 (31.5%)	1 (24.1%)	2 (22.3%)	3 (20.8%)	4 (20.1%)	5 (15.5%)	6 (14.7%)	7 (14.5%)
HN44	0.0	24.57	9.18	31.83	0.06	14.96	20.69	161.50
HN65	0.0	10.77	43.36	7.08	2.82	31.16	3.79	122.80

*The chart shows the relative increase rate on the next day compared with the previous day.*

## Discussion

Under prolonged limited water availability, plants undergo morphological changes aimed to cope with the adverse effects of stress (Liu et al., 2007). Additionally, MDA content generally increases with stress and is low in drought-resistant varieties. Therefore, MDA content was used as an indicator of membrane lipid peroxidation to reflect the ability of plants to respond to adverse conditions, such as drought (Ru et al., 2006). Previous studies have shown that corn and soybean MDA levels increase as drought intensity and duration increase (Ge et al., 2005; Bu et al., 2009; Du et al., 2015). Mo and Zhai (2007) tested two different drought-tolerant soybean varieties under conditions of drought stress. The authors found that as drought intensity increased, the texture, membrane permeability, and MDA content gradually increased, and POD and CAT activities initially increased and subsequently decreased. These results are consistent with the results reported herein. Furthermore, this finding may be due to the low plasma membrane permeability and MDA content of drought-resistant varieties compared with drought-susceptible varieties and the high CAT and POD activities of drought-resistant varieties. This finding differs from the results of this experiment and may be explained by differences in planting methods, varieties, and environmental conditions. Plants can reduce transpirational water loss by reducing leaf area under drought stress. Some studies have shown that drought stress can alter cellular membrane structure in soybean cells, induce cell membrane damage, increase membrane permeability, and induce the accumulation of a large number of biofree radicals, which will affect the normal function of cells, thereby affecting the normal physiological activities of soybean. These findings are consistent with the increase in MDA content observed in our experiment by withholding water.

Jiang et al. (2019) studied the effects of drought stress on MDA content and the activity of three antioxidant enzymes in the leaves of pecan seedlings. Under mild drought stress, the antioxidant activity of thin-shell walnut plants increased, and the MDA content gradually increased by self-regulation and adaptation mechanisms. Similarly, Rui et al. (2013) showed that with increasing drought duration, MDA content in seedlings increased, MDA levels and membrane permeability in the early stage of stress decreased slightly, and late stage increased significantly. Gao et al. (2016) showed that SOD, POD, and CAT activity and T-AOC in soybean leaves first increased and then decreased with processing time. During the treatment period, the indicators of the drought-resistant variety HN44 were present at higher levels compared with those from the drought-sensitive variety HN65. The change in T-AOC is different from the experimental results, which may be due to the short number of treatment days in this experiment.

Osmotically active molecules, such as proline, soluble sugars, and soluble proteins, reduce cell permeability under stress and maintain inorganic ion concentrations to ensure normal plant growth. Thus, for example, Wang Y. P. et al. (2014) showed that proline levels in soybean increased significantly during drought stress, and the number of varieties with high drought tolerance increased significantly compared to those with low drought tolerance. Consistently, while subjecting soybean seedlings to a period of drought stress, Gao et al. (2009) observed that, as the duration of drought increased, proline content increased. These results are consistent with the results of our experiment. Drought tolerance screening studies have shown that increased soluble sugar content in crops during drought can reduce cell permeability in the plant body and maintain normal metabolism, thus improving crop drought tolerance (Wang, 2010). Consistently, Zhuang et al. (2020) found that drought stress increased the proline and soluble sugar levels in corn leaves; furthermore, the greater the stress intensity, the higher the increase. This finding is similar to the results of our own experiment. Tan et al. (2008) showed that the soluble protein levels in a single peak curve, as observed in wheat seedlings, increased as drought intensity increased.

Li (2019) believed that drought can significantly change root development and related physiological processes, thereby affecting soybean yield. Irrigation can increase the soybean yield under dry conditions. The use of irrigation during critical periods of high water demand is an important method for increasing crop productivity (Zou et al., 2012). Therefore, in the future, drought resistance research on soybeans should focus the following aspects: (1) relevant departments should make advanced and accurate forecasts and provide early warnings of drought-prone and rainy areas, and different resistance measures and methods should be adopted according to the forecast to achieve disaster prevention and mitigation; (2) sowing of locally suitable varieties and the adoption of water-saving irrigation systems, such as sprinkle or drip irrigation, should be promoted in regions of arid farmlands; and (3) plant growth regulators should be applied to improve crop resilience (Wang et al., 2020).

## Conclusion

Soybean leaves removed ROS by accumulating osmotically active molecules and upregulating antioxidase activities to maintain the stability of the cell membrane and minimize drought stress-induced potential damage. The extent of soybean antioxidant activity and the T-AOC increased faster at soil moisture contents of at least 15.5%; furthermore, drought resistance of HN44 was consistently higher than that of HN65. The ROS removal system in soybean leaves was severely damaged at soil moisture contents below 15.5%. This finding demonstrates that under severe water deficit, stress injury is irreversible, and there is a certain threshold for the defense and self-repair ability of protective enzymes. Therefore, under black soil cultivation, the threshold soil moisture content during soybean flowering period was determined to be 15.5%.

## Data Availability Statement

The original contributions presented in the study are included in the article/supplementary material, further inquiries can be directed to the corresponding author/s.

## Author Contributions

XYW, ZW, and QZ wrote the section of the manuscript. XW and SS contributed to data curation and visualization. SD contributed to manuscript revision. All authors approved the submitted version.

## Conflict of Interest

The authors declare that the research was conducted in the absence of any commercial or financial relationships that could be construed as a potential conflict of interest.

## Publisher’s Note

All claims expressed in this article are solely those of the authors and do not necessarily represent those of their affiliated organizations, or those of the publisher, the editors and the reviewers. Any product that may be evaluated in this article, or claim that may be made by its manufacturer, is not guaranteed or endorsed by the publisher.
